# Integration of social determinants of health information within the primary care electronic health record: a systematic review of patient perspectives and experiences

**DOI:** 10.3399/BJGPO.2023.0155

**Published:** 2024-01-24

**Authors:** Nicolle Marianne Arroyave Caicedo, Emma Parry, Nazan Arslan, Sophie Park

**Affiliations:** 1 Department of Primary Care and Population Health, University College London, London, UK; 2 School of Medicine, Keele University, Staffordshire, UK

**Keywords:** social determinants of health, screening, primary healthcare, electronic health records

## Abstract

**Background:**

Social determinants of health (SDOH) are the non-medical factors that impact health. Although geographical measures of deprivation are used, individual measures of social risk could identify those most at risk and generate more personalised care and targeted referrals to community resources. We know SDOH are important to health care, but it is not yet known whether their collection via the electronic health record (EHR) is acceptable and useful from the patient perspective.

**Aim:**

To synthesise relevant literature to explore patient perspectives on integrating information about SDOH into primary care EHRs, and the opportunities and challenges of its implementation in a general practice setting.

**Design & setting:**

Systematic review of primary care-based qualitative and mixed-method studies using thematic framework analysis.

**Method:**

Key databases were searched for articles reporting patient perspectives of SDOH collection within the primary care EHR. Qualitative and mixed-methods studies written in English were included. A framework analysis was conducted to identify themes.

**Results:**

From 14 included studies, the following five main themes were identified: rationale for SDOH screening and the anticipated outcomes; impact of the provider–patient relationship on patient perceptions; data, which included privacy concerns; screening process and referral; and recommendations for future research.

**Conclusion:**

Integration of information on SDOH into the EHR appears acceptable to patients. This review has added to the discussion of whether and how to implement SDOH screening and referral programmes into UK primary care systems.

## How this fits in

Existing literature suggests that individual SDOH data, integrated into the EHR, is a better indicator of patients' needs compared with geographical data and that the primary care environment is a potential space for SDOH–EHR integration. This study synthesises and examines patient perspectives towards SDOH–EHR integration in primary care including: the perceived factors that facilitate patient acceptability (for example, pre-existing provider–patient relationship); the perceived anticipated benefits of SDOH–EHR integration (for example, the potential use of the data to tailor health interventions), and perceived challenges of integrating SDOH into the EHR (for example, poor availability of resources to manage social risk factors); and recommendations for SDOH–EHR implementation (for example, explaining its purpose clearly to patients).

## Introduction

Health inequalities in England are increasing.^1,2^ Social determinants of health (SDOH) refers to the non-medical factors that impact health,^2,3^ such as income, housing, and food insecurity.^4^ The relationship between SDOH and health has encouraged interventions tackling inequalities such as SDOH screening.^5^

SDOH screening and referral programmes have the potential to improve health care.^6^ Incorporating SDOH data into the electronic health record (EHR), as recommended in the US,^7^ could facilitate more accurate measurements of social risk and provide knowledge to identify and target 'susceptible' groups.^8,9^

Despite geographical data being used in the UK to measure deprivation,^10^ *'living in a deprived area is not the same as being deprived'* and vice versa.^11^ In studies integrating SDOH data into the EHR, individual rather than area-level data better predicted patient outcomes and needs.^9^ Individual measures could better identify those most at risk, leading to better personalised care and targeted referrals to community resources.^12–15^ Some suggest primary care is well positioned to collect this individual-level data,^16–18^ owing to the continuity of care^19^ and gatekeeper role of GPs, while emergency care could also be appropriate for some patients.^19^

Doctors have mixed views about SDOH screening. Some feel ill equipped to intervene on SDOH^20–22^ and feel they have insufficient time to address them.^22^ Others fear disruptions to workflow,^22^ being ‘overworked’,^23^ and unrealistic expectations,^24^ which might worsen the provider–patient relationship.^22^ On the other hand, some believe it will bring greater job satisfaction,^22^ a better perception of healthcare quality,^22^ and a deeper understanding of their patients.^12,14^

Existing literature has focused on screening feasibility,^12^ SDOH interventions,^24,25^ clinician acceptability,^12–14,26,27^ and patient acceptability.^28,29^ Patient acceptability of SDOH screening has been demonstrated via low refusal rates to screening,^30,31^ and quantitative survey responses to questions asking how comfortable or satisfied patients were with screening.^16,31–34^ However, no review has synthesised patient perspectives about merging SDOH within the EHR.

The aims of this review are to explore patient experiences, including the opportunities and challenges associated with integrating SDOH screening into the EHR in the context of primary care in the UK.

## Method

### Data sources

We searched MEDLINE, Embase, CINAHL, Cochrane Library, Scopus, and Web of Science, from January 2002–February 2022. The search terms (Supplementary Table S1) were piloted in MEDLINE and key stakeholders were consulted to ensure the search captured key papers. Our search comprised terms on SDOH, EHR, primary care, and patient perspectives. Government websites, such as the Office for Health Improvement and Disparities, were used to search for grey literature, using the search terms 'social determinants of health' and 'electronic record'.

### Inclusion and exclusion criteria

The inclusion criteria included the following: qualitative or mixed-methods papers; studies based in a primary care setting; papers written in the English language; studies that focused on the views and experiences of patients, which mentioned SDOH collection and primary care EHR. The exclusion criteria included having no patient perspectives or feedback, or studies that focused on secondary care settings.

### Selection process and data extraction

**Figure 1.**
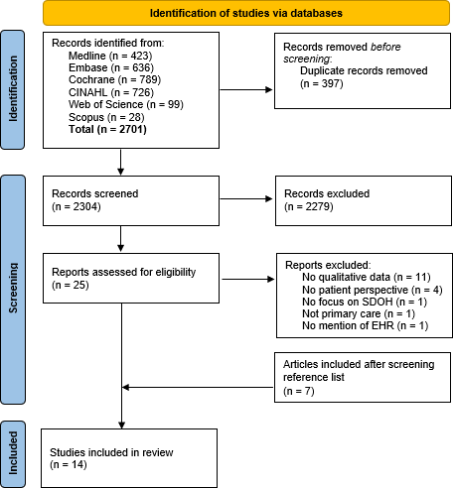
PRISMA flowchart.^62^ EHR = electronic health record. SDOH = social determinants of health

Title and abstract screening was undertaken by the first author, with 10% randomly screened by another author independently; disagreements were resolved by discussion. Full texts were assessed for inclusion by the first author and 10% of these were randomly screened by a second author. Disputes were resolved by a third reviewer. References from included studies were screened to identify any relevant literature. Figure 1 shows the Preferred Reporting Items for Systematic Reviews and Meta-Analyses (PRISMA) flow diagram. Data were extracted using an Excel spreadsheet to organise and manage sources.

### Data analysis

A thematic analysis was used to synthesise the data.^35^ The first reviewer (NA) conducted line-by-line coding of each included study using NVivo 20.^36^ All study members looked at similarities and differences between codes to group them together. Following this, three reviewers inferred the barriers and facilitators from the grouped codes independently and then as a group. Following group discussions, the overall themes began to emerge. This process continued until the overall themes that emerged explained all of the initial descriptive or sub-themes.^37^ The process was both deductive, to address the review questions, and inductive, to iteratively respond to the included data and ensure the review was relevant and representative of the included literature. NA kept a reflexive log during the analysis stage.

### Quality assessment

Critical Appraisal Skills Programme (CASP) tools were used to critically evaluate texts. Appraisal was not used to exclude papers (Supplementary Table 2).

### Patient and public involvement and engagement

Patient and public involvement and engagement (PPIE) representatives were recruited from an expert-by-experience panel at University College London. They were involved in the analysis and interpretation of data by reviewing the emergent themes and checking the researcher’s understanding of the data.

## Results

The literature search identified 2701 papers, 397 duplicates were removed, 2279 were removed at title and abstract screening, 18 were excluded at full-text review, and a further seven were included at citation screening. This resulted in 14 studies being included in the analysis (Figure 1).

The design of the 14 studies included the following (some studies used more than one element): one-to-one interviews (*n* = 8);^38–45^ surveys (*n* = 4);^42,46–48^ focus groups (*n* = 5);^40–42,49,50^ and a systematic review (*n* = 1).^51^ Eleven studies were based in the US,^38–44,46,47,49,50^ two in Canada,^45,48^ and one in the UK^51^ (See Supplementary Table S3).

### Quality assessment

The main limitations in these studies was the possible effect of social desirability bias^41^ and the limited diversity of patient groups, limiting the perspectives of those with additional discrimination, language, or immigration barriers^38,40,41,44,45^ and of patients who did not feel comfortable sharing their SDOH information^44,45,50^ (See Supplementary Table S2).

### Themes

From the data the following five overall themes emerged: rationale for SDOH screening and the anticipated outcomes; impact of the provider–patient relationship on patient perceptions; data; screening process and referral; and recommendations for future research. Although these are presented as five separate themes, there were some overlapping concepts. We have presented these separately for ease of understanding. Illustrative quotes for each theme can be found in Supplementary Table S4.

### Rationale for SDOH screening and the anticipated outcomes

#### Importance of screening for SDOH

Participants described the importance of SDOH screening, the high prevalence of social risk factors,^39^ personal experiences of SDOH,^39^ and SDOH’s impact on mental and physical health,^39,42,44,45^ family and friends.^44^ Participants made connections between SDOH and health; for example, the effect of food insecurity on diet-related illnesses, poor housing conditions on asthma, and SDOH-related stress on health.^39^

Some participants described how they normally avoid talking about SDOH with their doctor,^40,41^ owing to the stigma associated with asking for assistance,^41^ therefore they appreciated doctors initiating the discussion.^40,41,50^ Participants believed that by sharing SDOH information, the care team would gain a greater understanding of their health, the environment they live in (for example, a smoking household) and how it affects health (for example, asthma),^39^ which could improve their quality of care,^41,42,48,49^ and facilitate diagnoses previously missed without the social background.^39^

#### Improvements in personalised care

Some participants highlighted how doctors could use their SDOH information; for example, in tailoring interventions,^41,42,48^ and referring^41,42^ to relevant resources.

For those who had undergone SDOH screening, participants felt their access to resources increased or had the potential to increase.^40,42,50^ Some were connected to community services; given resources, such as baby formula; and received information about subsidised housing and food banks.^50^

#### Long-term use of SDOH data

Participants were more likely to find the process of screening acceptable if they believed it had positive long-term benefits.^42^ Some highlighted the opportunity to use EHR data to measure and identify health inequalities,^48,49^ which could be used to draw attention to key issues that *'perpetuate individual level social needs'*,^38^ or target '*at-risk'* populations.^48,49^ Participants were receptive to the idea of helping others by sharing their own SDOH data.^38^

The discussions and data could also raise awareness of the broader social issues,^44^ help advocate for community services that need development,^38^ and support health promotion interventions.^48^

### Impact of the provider–patient relationship

#### Rapport with healthcare professionals

Having an established and amicable relationship with a clinician or provider was a key facilitator to acceptability of SDOH screening.^38–42,44,50^

Behaviours that contributed to rapport were as follows: respectfully listening to the participant;^38,41,50^ showing empathy^39,41^ and compassion;^39^ being approachable;^39,50^ being knowledgeable about the available resources;^39^ sharing decision-making with the participant;^41^ recognising the strengths of a participant’s self-care efforts;^50^ a non-judgemental approach;^38^ and being reassuring.^44^

Participants were less satisfied if the doctor’s approach was impersonal,^39,42,50^ or paternalistic,^38^ which hindered participant participation^39^ and made adherence to plans more difficult.^42^

Some participants left with a more positive view of the doctor,^38–42,44,50,51^ feeling *'cared for'* ^38,39,44,50^ *'as a whole'*,^39,41^ which they thought strengthened their relationship^39–41^ and improved communication.^40^

#### Screening in primary care

Participant acceptability towards the process was higher when they believed the primary care setting was an appropriate place for SDOH screening.^39,44,47,50^ Some viewed the setting to be well-positioned,^39,47,50^ because of the longitudinal rapport,^47^ and because they perceived it as a *'safe environment*',^39,50^ *'where people help people'*,^39^ encouraging them to open up.^50^

On the other hand, some believed that dealing with SDOH was outside the remit of primary care.^39,50^ Some believed this was true of both screening for and resolving social needs,^50^ while others believed only the resolution was beyond their scope.^39^ Reasons for this included beliefs that primary care staff *'were not adequately trained or equipped to solve social issues*'.^39^

Mistrust in the healthcare system was also a barrier,^41,50,51^ especially in cases where participants had previously experienced discrimination or racism.^41^

#### Fear of referral

The fear of being referred to child protection services made some people reluctant to share their SDOH information,^38,44,50^ fearing they would be *'deemed an unfit parent and lose* [their child] *for not being able to provide better'*.^38^ Similarly, people were hesitant sharing information with someone they believed *'has the power*' to make calls affecting their immigration status.^38,41,50^

#### Proposed recommendations

Papers commonly recommended a clear explanation of the screening’s purpose and anticipated data usage.^38,39,41,42,44,49^ The link between SDOH and health should be explained^38,41^ as well as the potential benefits.^38,44^ Patients should be reassured they are not being singled out,^38,39^ and will not be negatively impacted by their results,^38,44^ and the privacy of their data^38^ should be explained.

Doctors were advised to acknowledge patients’ feelings of shame,^44^ as well as the discrimination^41^ and barriers to assistance they may face.^38^

Suggestions for provider conduct included empathy and compassion,^39^ being non-judgemental,^42,44^ reflecting^41^ and being honest that it may not be possible to provide resources beyond what the patient has already found.^38^

Authors suggested training staff on shared decision-making,^41^ non-judgemental communication,^44^ SDOH,^51^ and how to discuss SDOH issues sensitively.^50,51^ Practices are recommended to implement quality improvement strategies,^44,50^ and obtain buy-in from leadership, administrators, and clinicians, as well as support from SDOH champions, IT officers, and EHR experts.^49^ Institutional support would help with approval from stakeholders,^41^ who should be consulted at an early stage to clarify workflows and data usage.^49^ Patients or community representatives should also be consulted,^48,49,51^ when making decisions about the screening method and evaluating effectiveness.

### Data

#### Data storage in the EHR

Being comfortable with their primary care team accessing their EHR data^41^ and believing EHR integration was beneficial^39,41^ in providing team-based care,^39^ were facilitators to acceptability.

#### Confidentiality

Some participants were more concerned with EHR documentation of SDOH screening than the screening itself^47^ and confidentiality was a major concern.^38–40,42,51^ Worries were based on feeling shame if community members found out about their social needs^38^ and being avoided if deemed more challenging by clinicians owing to their social risk factors.^51^ Others feared the process would expose them to discrimination,^38–40,42,45,48^ concerned that those accessing their data would judge them,^38–40,42^ leading to changes in the standard of care,^48^ or loss of services.^45^

#### Data quality

Some participants had doubts about how truthful participants would be^40,44,45,51^ owing to fear of judgement,^44,51^ mistrust,^51^ and confusion with the questions.^45^

#### Proposed recommendations

Screening for SDOH should also occur regularly,^38,40^ with frequency dependent on the patient,^40^ balancing the benefits with the '*burden patients may feel from frequent assessments that ask the same sensitive questions'*.^40^ Some suggestions were screening every 6 months or every visit.^40^

The integration of SDOH data with the EHR was supported,^44,48,49^ as it could facilitate routine screening,^44^ avoid manual data entry^48^ and be linked with outcome data,^49^ but areas for improvement were identified, including solving issues entering, accessing, and sharing EHR data.^51^

Some said there should be privacy protocols in place,^39^ processes to obtain patient consent for data,^42,49^ and buy-in from a privacy officer.^49^

### Screening process and referral

#### Views on screening

Participants were generally satisfied with the screening process,^39,42,43,45,48^ feeling grateful,^39^ relieved, or reassured^39,42^ that clinicians were asking about social needs.

Some participants explained how the discussion alleviated shame about experiencing social risk factors^44^ and asking for help.^41,42^ Others explained how it made them realise that others also struggled with social risk factors^44^ and they felt less isolated.^39,44,47^

For some, the questions were *'too personal*', especially finance questions.^40,48^ They also experienced shame^38,40,44^ about experiencing social risk factors,^40,44^ which made some *'feel like a failure'*.^44^ Some felt distress,^40,44^ because of the *'frustration and helplessness'* they experienced,^44^ and because screening triggered *'unpleasant memories'*,^40^ making disclosure of information more challenging.^40^

#### Referral process

The process was perceived to be more helpful if the clinic promptly and proactively linked people to resources.^37,42^

Participants deemed poor access to resources a key barrier.^41,42,44,46,51^ They sometimes experienced not hearing back from services,^46^ long application processes,^41^ having to reapply to services several times,^46^ receiving incorrect information,^41^ and scheduling difficulties.^42^ Resources were seen as being in short supply, underfunded,^51^ and unable to deal with the '*Pandora’s box'* of social needs that emerged from screening.^41^ Some papers reported concerns that without resource availability, asking participants about social needs was inappropriate.^51^

#### Practicalities of SDOH screening

Patients or caregivers filled out screening surveys in nine studies,^38–40,42,45–48,50^ clinicians completed screening in two studies,^44,50^ and alternative staff members screened patients for SDOH in four studies^38,41–43^ (see supplementary Table S5).

Participants outlined their thoughts on self-reported screening. Key advantages were feeling that they would not be judged^40^ and the universality of screening.^48^ Participants preferred data collection to be *'simple to understand'*,^48^ and available in different languages.^50^ Downsides were the difficulties reading and filling out electronic surveys, especially for older participants,^40,48^ the fact completion in the waiting room depended on how soon they were called into their appointment,^48^ and how feeling rushed could affect the thoroughness of answers.^40^ Some were worried that time constraints of a consultation would also make it difficult to discuss social risk factors.^40^

#### Who should undertake the screening?

Some described self-reported questionnaires as *'burdensome'* ^40,42,48^ or stressful as the responsibility was on them.^40^

Although participants were comfortable with either doctors or other staff carrying out the screening,^51^ some preferred nurses or social workers, who they felt were more empathetic and less *'medical'* than doctors.^50^

Participants felt that quality of care could worsen^45,51^ if screening were to be carried out *'at the expense of clinical tasks*', or might *'overwork'* doctors.^51^

Table 1 depicts the participant-reported advantages and disadvantages of patient-reported and staff-led screening.

**Table 1.** Summary of patient reported advantages and disadvantages of patient-reported and staff-led screening

Self-reported survey		Consultation with doctor or staff	
Pros	Cons	Pros	Cons
Would not feel judged^40^	Doesn't allow collection of factors that warrant a timely reaction^49^	Chance to build rapport^38^	Not realistic owing to time constraints^38^
Negates concerns of being overheard^40^	Information may be influenced by social desirability bias, stigma, or self-interest^51^	Chance to explain purpose^38^ and questions^40^	Patients may *'feel judged'* more^40^
*'More time to think*' about answers^40^	Has limited communication about survey purpose,^38^ and limited explanation of questions for those with issues understanding^40^	More effective method to elicit sensitive information owing to personability.^41^ *'When they ask you, you feel more comfortable*'^50^	Risk of being overheard^40^
More honest responses^40,44^	Too many tasks for patient to complete^40,42,48^	More accurate responses^40^	

#### Accuracy of data

Some participants were concerned about the accuracy of data, particularly income, which can fluctuate, and severance pay and retirement incomes, which could be misleading as reflections of health.^45^

There were concerns about the ability to update data and how often this would occur,^40,42,48,51^ with one participant saying, *'once it’s in there it’s not getting out and I don’t know how to update it'* ^42^ and another saying that screening annually was insufficient.^40^

#### Proposed recommendations

Papers suggested screening should lead to *'actionable information'*,^42^ and that there must be ways to analyse and react to data^49^ and follow-up appointments to ensure needs are addressed.^41,42^

Recommended actions were giving information about services,^39,44,46,51^ referring patients to community organisations,^39,40^ and helping patients apply to them.^44^ Some patients did not expect primary care to resolve their social needs, just to be aware of them.^39^

To implement these actions, relevant resources need to be identified,^49^ staff need to be knowledgeable about them,^51^ and actions need to reflect their availability,^41^ not outpacing their capacity.^42^

Reflecting on the fact that actionability is limited by the resource availability, many encouraged increased efforts at a policy level to address the lack of funding, availability, and effectiveness of resources.^38,40,41^

There were suggestions to clarify the survey content, such as to not use unfamiliar words,^42,48^ reduce repetition between questions,^42^ expand the lowest income range to make patients comfortable answering,^48^ and use a single-question screener such as *'Do you (ever) have difficulty making ends meet at the end of the month?*'.^51^ People with low literacy could have staff to assist patients with electronic surveys.^40^

It was also proposed that social risk factors that required a *'timely reaction'* were only collected within consultations, not in remote surveys^49^ and that screening should not be compulsory.^50^

### Recommendations for future research

Priorities identified in the studies included future research that should focus on the following: (i) how often screening should take place;^42,48,51^ (ii) how EHR tools or text messages can be used to prompt repeat surveys;^38,48^ (iii) which is the most effective and most acceptable screening method;^42^ (iv) what the proven benefits to SDOH–EHR integration are;^45,51^ (v) what the perceptions are of patients who did not feel comfortable being interviewed or disclosing SDOH information.^44^

## Discussion

### Summary

Support for SDOH screening was mostly encouraging. Screening was thought to be important to help healthcare professionals understand the context of someone’s health and the complex interplay of environmental factors. People had more confidence in the screening process if they had trust in their healthcare providers, understood the reason for screening, and there was an actionable outcome. Opinions were mixed on whether primary care was the most suitable place for SDOH screening. Some people were particularly concerned about confidentiality and who would have access to their data.

### Strengths and limitations

Many themes were consistent across the studies, suggesting the dependability and transferability of ﬁndings. Key strengths include an eligibility criterion that included both mixed-methods and qualitative research, and the collaboration of PPIE representatives. Themes were developed and reviewed by three authors. The authors were cognisant of their professional backgrounds and how this may have affected data collection and interpretation through the research process through keeping logs and discussions at team meetings. The PPIE members were involved at the analysis and interpretation stage to strengthen findings through triangulation.

The limitations include the applicability of results, as only one paper was set in the UK and there was a lack of diversity of reported participants in papers. Studies were not excluded based on quality assessment as we wanted to ensure we included articles with a broad range of patient views; however, this may have impacted on the robustness of the review. Because this is an innovative field of research, the terminology and search engine keywords are still not well developed, leading to possible incomplete retrieval of all relevant papers. Therefore, the research team decided that citation screening and stakeholder feedback would be included.

### Comparison with existing literature

The broader literature on patient perspectives of SDOH identified similar anticipated uses for SDOH screening such as tailored referrals,^52^ and similar concerns, including the fear of judgement.^34^ A key finding was that acceptability was based on whether patients understood the purpose of the screening, and thus clearly explaining the purpose to patients was recommended.^34,53,54^

A key finding in this study was patient concerns of confidentiality with social risk factors being included in the EHR. In contrast, other studies thought this would be convenient as new doctors would have easier access to their data.^55,56^ Accuracy of data was also important, and our review has added to previous literature by highlighting the challenge of keeping records up to date.^38,40,57^ Of note, patient access to their data online has led to increased patient engagement,^58^ and may offer new opportunities and challenges if SDOH screening is undertaken in primary care.

Our review highlighted the mixed preferences of screening method, which contrasts with a randomised trial that showed greater disclosure of social risk factors in self-reported methods rather than face-to-face methods.^59^

Despite our findings showing that screening was more acceptable if there was an actionable outcome — for example, referral to local resources — previous studies have demonstrated that not all patients would like additional information, even if living with negative effects of social risk factors.^60^ This highlights the importance of offering resources but not making these compulsory to engage with.

### Implications for research and practice

Despite considerable data pertaining to the SDOH screening process, there was a lack of in-depth data focusing on its integration into the EHR. The lack of evidence showing long-term benefits limits the ability to recommend its implementation. In addition, it is important to capture the perspectives of UK clinicians and patients, which may differ from the predominant US perspectives in the review. Questions remain on which SDOH domains to include in screening. Pinto *et al* outlined the rationale for each question;^48^ however, these domains need to be assessed for compatibility in the UK. The literature explored patient perspectives of universal screening approaches; however, other approaches may also be practicable for patients and practices, taking into consideration their available time and resources. Other possibilities of course exist, including more agile or responsive recording of data within clinical encounters if and when identified or needed. However, some patients did report the value of universal screening as less judgemental.^38^

This review has highlighted the importance of different stakeholder perspectives and that *'*[general practice] *reform must be grounded in a recognition of what matters most to patients and practitioners: quality, convenience, choice, and continuity'*.^61^ Key to the debate on whether SDOH screening should be undertaken in primary care is implications on workload, whether healthcare professionals feel trained to deal with social issues and whether resources are available once a need is identified.^19,21,22^ We also need to ask whether primary care is the correct place for this to take place. Screening has implications for public health, local authorities, and government, so it is important to ensure all stakeholders are involved in all discussions along with patients.

In conclusion, this review has added to the global discussion of whether and how to implement SDOH screening and referral programmes into the EHR, based on patient experiences and preferences. Patient perspectives are crucial to embed into future studies and to consider in the future organisation and design of SDOH–EHR initiatives. It is as yet unknown how it could be best implemented in a UK-based workflow and indeed whether primary care is the best place to screen for social risk; however, integrating information on SDOH appears acceptable to patients.

## Trial registration number

The systematic review was registered on PROSPERO (Ref. CRD42022321472), in accordance with PRISMA statement for systematic reviews.

## Data

The data relied for the conclusions of this article is available in the supplementary materials.
